# Protein Tertiary Structure by Crosslinking/Mass Spectrometry

**DOI:** 10.1016/j.tibs.2017.12.006

**Published:** 2018-03

**Authors:** Michael Schneider, Adam Belsom, Juri Rappsilber

**Affiliations:** 1 Chair of Bioanalytics, Institute of Biotechnology, Technische Universität Berlin, 13355 Berlin, Germany; 2 Wellcome Centre for Cell Biology, University of Edinburgh, Edinburgh EH9 3BF, United Kingdom

## Abstract

Observing the structures of proteins within the cell and tracking structural changes under different cellular conditions are the ultimate challenges for structural biology. This, however, requires an experimental technique that can generate sufficient data for structure determination and is applicable in the native environment of proteins. Crosslinking/mass spectrometry (CLMS) and protein structure determination have recently advanced to meet these requirements and crosslinking-driven *de novo* structure determination in native environments is now possible. In this opinion article, we highlight recent successes in the field of CLMS with protein structure modeling and challenges it still holds.

## Highlights

The earliest structural studies on proteins using crosslinking/mass spectrometry aimed to elucidate their tertiary three-dimensional structure.

Tertiary structure modeling using crosslinking fell out of favor for almost two decades because crosslink data were not informative to aid structure modeling.

Two game-changing trends emerged: using short-range crosslinkers that capture relevant modeling information and high-density crosslinking.

High-density crosslinking uses unspecific crosslinkers to dramatically increase crosslink numbers.

In addition, computational structure modeling methods made significant progress in exploiting CLMS data.

The combination of high-density crosslinking and computational structure modeling enables the elucidation of tertiary protein structure in native environments.

This sidesteps the key limitation of today’s structure determination methods, which are unable (except for a few, specialized methods) to probe the structure of proteins in cell lysates or even intact cells.

## A New Age of Protein Structure Analysis

We can better understand the function of a protein on a molecular and mechanistic level by analyzing its structure. Structural information boosts our ability to engineer proteins, design drugs, and comprehend the molecular basis of life. Thus, researchers developed several scientific methods to determine protein structure; structure determination methods and solved structures have earned – at least in part – six Nobel prizes in the past twenty years (1997, 2003, 2006, 2009, 2012, and 2017^1^). The main methods for solving structures at atomic resolution are X-ray crystallography, nuclear magnetic resonance (NMR) spectroscopy, and cryo-electron microscopy. Despite their indisputable progress, each of these methods has specific limitations. X-ray crystallography relies on the ability of proteins to form crystals with specific properties. NMR can probe protein structure in solution, but is limited to small proteins. X-ray crystallography and NMR both require highly purified protein. Cryo-electron microscopy can resolve the structure of protein assemblies that are typically large (>200 kDa), homogenous, and rigid, and is increasingly able to resolve the structure of individual proteins in favorable cases [1].

The three main structure determination techniques share the same general issues: they tend to study proteins in artificial environments and provide often only partial structures. These artificial environments do not resemble the cellular environments in which proteins function. Thus, the function we deduce from structures might be artifactual and divorced from biology. Researchers are, therefore, developing methods, including in-cell NMR 2, 3 and cryo-electron tomography [4], to probe protein structures beyond this limitation: in nativelike environments, or even in the cell.

In this opinion article, we argue that the tertiary structure of proteins can be probed in native environments using crosslinking/mass spectrometry (CLMS). Thus, CLMS might overcome the key limitation of traditional structure determination techniques. We will first give a brief introduction to CLMS and then discuss computational structure modeling and how the combination of the two methods sometimes enables researchers to generate tertiary structure models. Please refer to Box 1, Box 2, Box 3 for a detailed introduction to CLMS and protein modeling. While the majority of literature in CLMS reports studies on modeling protein complexes 5, 6, 7, this opinion will focus on recent advances in tertiary structure modeling using crosslinking data (see Table 1 for a summary of recent approaches).

**Table 1:** Studies and Modeling Resources for Crosslink-Driven Protein Tertiary Structure Modeling

Study	Crosslinking/mass spectrometry	Data analysis	Protein structure modeling
Young *et al*. (2000) [10]	BS3 crosslinker with MALDI-postsource decay mass spectrometry	Automated Spectrum Assignment Program (ASAP)	Scoring of threaded models with crosslink constraints
Kahraman *et al*. (2011) [46]	Simulated crosslinks	Simulated crosslinks	XWalk algorithm for computing sSASD for crosslinks. Can be used for validation and visualization
Kahraman *et al*. (2013) [35]	Crosslink data from the literature	Crosslink data from the literature	Comparative modeling and *de novo* protocols using Rosetta [68]; XWalk [46] for validation
Hofmann *et al*. (2015) [49]	Simulated crosslinks	Simulated crosslinks	*De novo* modeling with fast arc length scoring of crosslinks for solvent-accessible surface approximation
Matthew Allen Bullock *et al*. (2016) [47]	Crosslink data from the literature [35]	Crosslink data from the literature [35]	JWalk algorithm for crosslink modeling using sSASD. Development of scoring metric that accounts for nonaccessible residues
Belsom *et al*. (2016) [18]	Sulfosuccinimidyl 4,4′-azipentanoate (sulfo-SDA) crosslinker with liquid chromatography–MS	Xi [27] for database search and XiFDR [33] for false discovery rate estimation	Guided model-based search [50] integrated into the Rosetta package [68]
Degiacomi *et al*. (2017) [48]	Crosslink data from the literature [35] and simulated crosslinks	Crosslink data from the literature [35] and simulated crosslinks	Crosslink modeling using shortest solvent-accessible distance and explicit modeling of protein flexibility (DynamXL)
Brodie *et al*. (2017) [64]	Several zero-length and short-range crosslinkers. Liquid chromatography–MS	Isotopically Coded Cleavable Cross-Linking Analysis Software Suite and Kojak [69]	Replica exchange discrete molecular dynamics

Box 1 **Crosslinking/Mass Spectrometry**

A crosslinking/mass spectrometry experiment has at least three experimental steps [8]: (i) incubating the protein or protein mixture with a crosslinking reagent, (ii) digesting the protein into peptides, and (iii) mass spectrometric analysis of the resulting peptide mix.

The crosslinker is a reagent with at least two functional groups (and a spacer between them) that react with the protein. During incubation, the crosslinker reacts with the protein and forms covalent bonds. In case of photo-crosslinking, the photoreactive groups need to be activated with UV light [18]. We can deduce the upper distance bound of the crosslinked residues, because the crosslinker reagents have a defined length: Two residues can react only if the distance of their reactive groups is within the length of the crosslinker. Thus, the reacted crosslinkers store spatial information. To access this spatial information, we must determine the crosslinked residue pairs.

This is facilitated by digestion of the protein, followed by mass spectrometry. Digestion cuts the protein into peptides. The most commonly used protease is trypsin, which cuts the sequence after lysine or arginine residues (if neither is followed by a proline). The crosslinks withstand digestion, which results in a mix of linear peptides (which are not crosslinked) and crosslinked peptide pairs.

In the next step, mass spectrometry identifies the crosslinked peptides. An online reverse-phase chromatography column separates the peptides by hydrophobicity and injects the sample continuously into the mass spectrometer. If the mass of the entire peptide pair + crosslinker would be unique, simple mass matching would be sufficient to pinpoint the different peptides. However, because many peptides overlap in mass, especially for complex samples, this is not sufficient. Instead, researchers use tandem mass spectrometry to access sequence information of the peptides to enable peptide identification. The mass spectrometer selects the most intense peaks (corresponding to most abundant peptides) during a mass scan (MS^1^). The selected peptides are fragmented in a fragmentation chamber. Different types of these fragmentation methods are available, but the most common are collision-induced dissociation and its high-energy variants (high-energy collision dissociation). Peptides collide with an inert gas, which breaks the peptide bonds. The resulting fragments are analyzed, resulting in a second mass spectrum (MS^2^). Because the fragmentation spectra contain more information about the sequence, spectra are later matched to the possible peptides and peptide pairs in database search. Note that other crosslinking and acquisition pipelines might use even more spectra acquisitions (MS*^n^*) [16].

Alt-text: Box 1

Box 2 **Database Search and False Discovery Rate Estimation**

After the mass spectra are recorded (see Box 1), the recorded spectra need to be matched to the crosslinked peptide pairs to pinpoint the crosslinked residues 27, 28, 29, 30. Because the sequence information of the fragmentation spectra is typically insufficient to directly read out the sequence *de novo*, most researchers employ a database search method. In addition to the recorded spectra, database search requires the sequences of the proteins contained in the sample as input. The algorithm *in silico* digests the peptides and generates the theoretical fragmentation spectra. These spectra are then ‘matched’ to the recorded fragmentation spectra.

There are many ways of matching and scoring the spectra, such as probabilistic analysis 27, 28 and cross-correlation [69]. This results in peptide spectrum matches (PSMs) in which the match of a peptide and a recorded mass spectrum is scored. A complicating factor of CLMS is that crosslinked peptide pairs need to be matched to the spectra. Thus, researches need to consider every possible peptide pair, which results in a large, quadratic (*n*^2^) search space. The approaches to cope with this search space complexity is beyond this article, but please refer to this review for more details [70].

The output of database search is a list of scored PSMs. One issue in interpreting the PSMs is that the score distribution of true and random sequences with the recorded spectra overlaps. Thus, it is difficult to decide on a score cutoff to separate true positive PSMs from false positives. A common approach to solve this dilemma is to use reversed or random ‘decoy’ sequences that are also matched to the spectra. Because we know that the decoys are false positives, we can use the score distribution to estimate the error rate (the so-called false-discovery rate) at a given score cutoff. This allows researchers to select a score cutoff at a controlled error rate 32, 33.

Alt-text: Box 2

Box 3 **Hybrid Structure Modeling**

Protein modeling is the set of computational techniques used to model the three-dimensional structure of a protein or protein complex. For tertiary structure modeling, there are two broad classes: comparative modeling and *de novo* structure prediction. Comparative modeling uses the sequence of the target protein to detect proteins with similar sequence in the protein structure data bank [40]. A subclass of comparative modeling, homology modeling, uses the homology assumption that proteins with similar sequence also have similar structure. The detected structure of a homologous protein in the PDB then serves as a template to build the target structure. In cases in which there is no structure of a homologous protein in the PDB, fold recognition (also called threading) is sometimes able to detect proteins with a similar fold but with low sequence similarity. Fold recognition methods employ a rich set of sequence-profile and structural features, often combined with probabilistic models, to detect a structure with a similar fold in the PDB 37, 38, 39.

If comparative modeling fails or a template is not available in the PDB, the protein must be modeled by *de novo* structure prediction [41]. *De novo* structure prediction folds the protein from the extended chain by searching the conformational space. Each conformation is evaluated by a score function that often contains physics- and statistics-based terms (although pure physics/statistics variants also exist).

*De novo* methods sample the conformational space by using Monte Carlo sampling 18, 41 or molecular dynamics [64]. From the resulting ensemble of structures, the native structure must be selected. This often involves clustering of the resulting structures or rescoring with sophisticated scoring functions [41].

Both types of approaches benefit from additional, experimental information. Methods that combine experimental information and computational structure modeling are called integrative or hybrid methods 14, 18, 49, 59, 64, 71. Experimental information imposes constraints on the structure and can be used to select the correct template if no clear match can be found, to steer conformational space search by adding the experimental constraints to the scoring function, and to select the final structure from the ensemble that is consistent with the experimental information [35].

The advantage of hybrid methods is that they enable the modeling of proteins for which not enough experimental information can be collected to determine the structure or that are too difficult to model with computational methods alone.

Alt-text: Box 3

## Crosslinking/Mass Spectrometry

**Figure 1:**
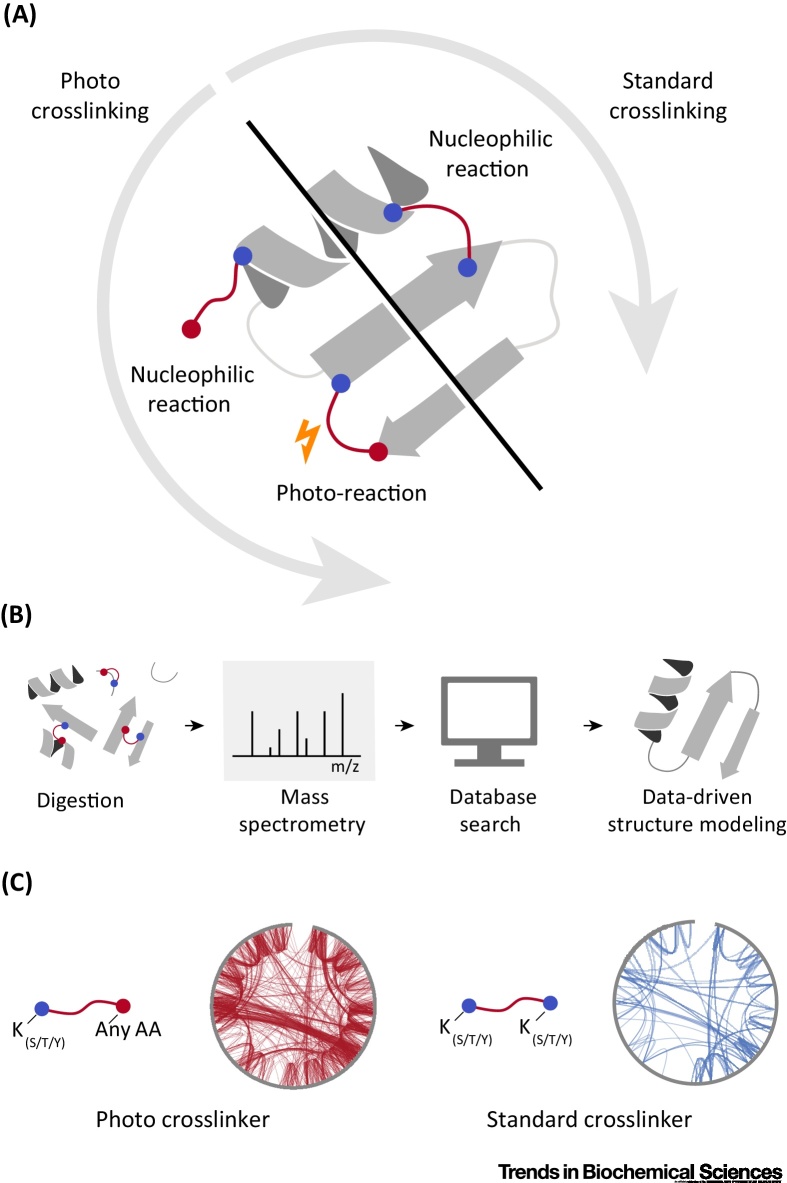
Overview of a Crosslinking Experiment for Protein Structure Determination. (A) As the first step in standard (homobifunctional) crosslinking, the crosslinker reacts with a specific reactive residue and then a second one to form a crosslink. Photo-crosslinking with photoactivatable reagents follows the same workflow. However, in the nucleophilic reaction step, only one side of the crosslinker reacts with the protein. The other side is activated by UV light and then reacts with the protein to form the crosslink. (B) The experimenter digests the protein using proteases (usually trypsin). The resulting peptides are then subjected to mass spectrometry. Specialized database search software reads out the crosslinks from the mass spectrometry data. The crosslinks then form the input to data-driven protein structure modeling. (C) Photo-crosslinkers such as sulfosuccinimidyl 4,4′-azipentanoate react on one side with lysine (and S/T/Y) and can react with any amino acid on the other side. This leads to a high crosslink density (the sequence of the protein is depicted by the circle; the crosslinks are shown as lines). These crosslinks can be leveraged for structural modeling. The reaction specificity of standard homobifunctional crosslinkers targets lysines (and S/T/Y residues to a lesser extent). This limits the density of the resulting crosslink network.

Crosslinkers act as molecular probes that introduce covalent links between amino acid residues in close proximity (Figure 1A) [8]. These links can then be read-out by MS following workflows that share many elements with standard proteomics applications that identify and quantify proteins: digestion of proteins into peptides, liquid chromatography–MS analysis, and subsequent database searches to identify the linked peptides (Figure 1B). Crosslinks provide three-dimensional information on individual protein structures and identify protein–protein interactions in protein complex assemblies and cellular networks 9, 10, 11, 12, 13, 14, 15, 16, 17, 18. Furthermore, conformational changes can be interrogated by quantifying the crosslinks that arise from different conformations of a protein or complex 19, 20, 21, 22. Importantly, CLMS can produce data in the native environment where the protein resides. This is possible because the crosslinkers can react under physiological conditions and once reacted, the protein can be denatured without losing the crosslinks and thus the structural information they encode.

The level of detail revealed by MS is typically low because crosslink data give sparse coverage of the 3D structural space. These sparse data are mostly caused by limitations of the most commonly used crosslinking chemistries. Standard approaches predominantly rely on amine-reactive *N*-hydroxysuccinimide esters specific to only lysine residues and protein N termini (although the free hydroxyl groups of serine, threonine, and tyrosine display some reactivity in peptides 23, 24, 25 and can account for 16% of crosslinked residues and 28% of crosslinks in a multiprotein complex [26]). This high specificity limits the potential combinations of crosslinked residues and so leads to few but abundant potential crosslinked peptides. This simplifies the analysis of MS data through specialized software, which matches the recorded spectra to all possible combinations of theoretical peptide pairs, modifications, and crosslink sites 27, 28, 29, 30. Current approaches also match inverted or shuffled ‘decoy’ sequences to the spectra to estimate the error of the identified crosslink, assuming that these decoy hits model the distribution of false positives 31, 32, 33. Sparse data from specific crosslinkers have proven highly valuable for studying protein complexes and networks 9, 11, 12, 13, 14, 15, 16, 17, 34, but less so on smaller scales where finer detail is desired 10, 35.

Higher crosslink data density could reveal these finer details and make detailed protein structure modeling viable. Photo-CLMS uses bifunctional, semispecific crosslinkers, which carry a specific group on one side and an unspecific group on the other side. This relaxes the strict residue specificity for crosslinking (to any N–H or C–H bond in proximity to a specific anchoring residue), and greatly increases obtainable data density (Figure 1C). However, questions remain as to how data from these experiments can be best analyzed, how data density can be further increased, and how these data can be best exploited.

## Computational Modeling of Protein Structure

Protein structure prediction is the discipline of predicting the structure of a protein from its sequence. There are two classes of protein structure prediction methods: comparative modeling and *de novo* modeling. Comparative modeling identifies related proteins that have structures in the PDB and uses these structures as templates to build the coordinates of the target structure. Comparative modeling can be further subclassed into homology modeling and fold recognition. In homology modeling, a homologous protein can usually be found in the PDB (i.e., the protein is solved in a different organism). Thus, the sequence similarity between the target and the template sequence is usually high and the homologous sequence can be identified by using sequence alignment tools such as Basic Local Alignment Search Tool (BLAST) [36]. If no template with high sequence similarity can be found in the PDB (usually because no homologous structure is solved), fold recognition can sometimes find proteins that have a related fold but are more distant in sequence space. Fold recognition methods can detect more distant folds because they use more sophisticated scoring metrics, based on sequence-profile and structural features, to measure the quality of a target–template alignment 37, 38, 39. If the sequence identity between the target and the template is high, comparative modeling can lead to highly accurate models [40]. However, since sequence identity correlates with model quality [40], many comparative models (especially when sequence identity is low) contain significant errors and it might even be difficult to select the correct fold. Regardless of the specific case, comparative modeling is only viable if a suitable template structure is available in the PDB.

If no template structure is available, the only applicable method is *de novo* modeling. *De novo* modeling mimics the folding process to some degree. These algorithms start from the unfolded chain and sample conformations to find the lowest energy structure. The most effective methods in this category use short fragment structures extracted from the PDB [41] to sample the conformational space. In addition, most methods use energy functions that are tuned to increase the gap between the native and all other conformations [41]. However, *de novo* modeling is routinely applied only to proteins up to 100 amino acids and, even then, is challenged by nonlocal residue–residue contacts (residue pairs that are close in space but not in sequence), which are often found in β-sheets. Additional information can push this boundary to proteins up to 300 amino acids and more complex topologies [42]. Computationally, evolutionary constraints provide such an additional information source for proteins with many homologous sequences. These are usually prokaryotic sequences due to the many prokaryotic sequencing projects [43]. Experimental data such as crosslinking can play a similar role in pushing the boundaries of size that can be modeled by providing distance constraints across the protein. Crosslinking stands out as a general experimental method due to (i) its modest sample requirements regarding the amount and purity of the protein; and (ii) allowing the protein to remain in solution in an environment that suits the needs of the protein rather than that of the technology.

## Strategies for Combining CLMS Data and Modeling

**Figure 2:**
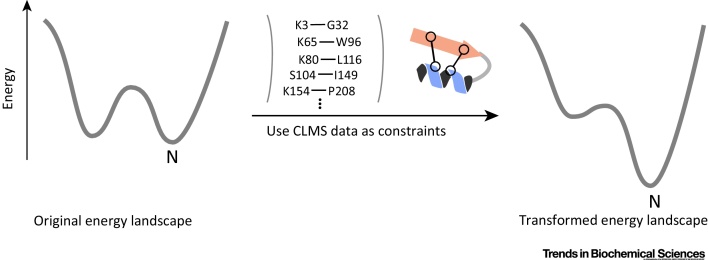
Effect of Crosslinking/Mass Spectrometry (CLMS) Data in Conformational Space Search. *De novo* protein structure modeling searches the conformational space of the protein for the lowest energy conformation, which usually coincides with the native structure. However, the energy landscape is rugged, and the energy of the native state might be close to the energy of other local minima. This makes search difficult because there might be no clear gradient toward the native structure. Using CLMS data as residue–residue constraints transforms the energy landscape by deepening the energy well of the native structure. This also makes the energy landscape less rugged and provides a gradient toward the native state. This makes it easier to search for the native conformation and therefore leads to more frequent sampling of nativelike structures in *de novo* structure modeling calculations.

Crosslinking data from standard crosslinkers alone are currently not sufficient to determine the structure of a protein. Likewise, as discussed, computational methods alone are often not able to model the structure of a protein without the use of templates. The combination of the two into a hybrid method, using CLMS data as distance constraints and computational methods to search the conformational space, is sometimes sufficient to enable more accurate modeling of protein structure, at least in favorable cases (Figure 2). Initially, effort on protein tertiary structure modeling focused on maximal use of very sparse crosslink data from specific standard crosslinkers.

Researchers have developed methods to cope with the sparseness (caused by highly selective crosslinking reagents) and low spatial resolution (because the Cα–Cα distance of crosslinked residues is the sum of the length of the side chains and the linker region of the crosslinker) of crosslinking structural constraints. Merkley *et al*. [44] investigated the upper distance bounds of CLMS constraints in molecular dynamics studies and suggested that an upper distance bound of 24–30 Å might be appropriate for the disuccinimidyl suberate (DSS)/bis(sulfosuccinimidyl) suberate (BS3) crosslinkers. To alleviate the issue of low spatial resolution, several modeling studies aimed to maximize the structural information from CLMS constraints, for instance, by mandating that the physical crosslink should be found along the protein surface and not penetrate the protein. Several groups developed algorithms to model this effect and compute the distance between crosslinked residues over the solvent-accessible surface of the protein model instead of computing the Euclidean distance between Cα atoms 45, 46, 47. XWalk and JWalk put the protein model into a grid and used breadth-first search to find the shortest solvent-accessible surface distance between crosslinked residues (sSASDs) 46, 47. These solvent-accessible distances can then be used in scoring schemes to measure whether crosslinks are satisfied in model structures. Using sSASD in scoring can improve structure selection from comparative and *de novo* modeling, which has been confirmed by Kahraman *et al*. [35]. Matthew Allen Bullock *et al*. [47] developed a new scoring scheme that uses sSASD and also penalizes crosslinks on nonaccessible residues, because buried residues should not be able to react with the soluble crosslinker. DynamXL explicitly accounts for protein flexibility for sSASD calculation and the authors show that accommodating flexibility in crosslink modeling improves the accuracy in protein docking [48]. Still, the drawback of sSASD to validate crosslinks in protein structures is its high computational cost, which prevents its use during the structure sampling phase and therefore cannot guide the search process. To efficiently use SASD as a part of the scoring function during *ab initio* calculations, Hofmann *et al*. [49] developed a faster crosslink modeling method by approximating the protein surface by the arc distance on a sphere. The authors found that using their representation of crosslink distance in scoring reduces the root mean square deviation (RMSD) by 1.0 Å on 2055 proteins in *de novo* modeling experiments.

**Figure 3:**
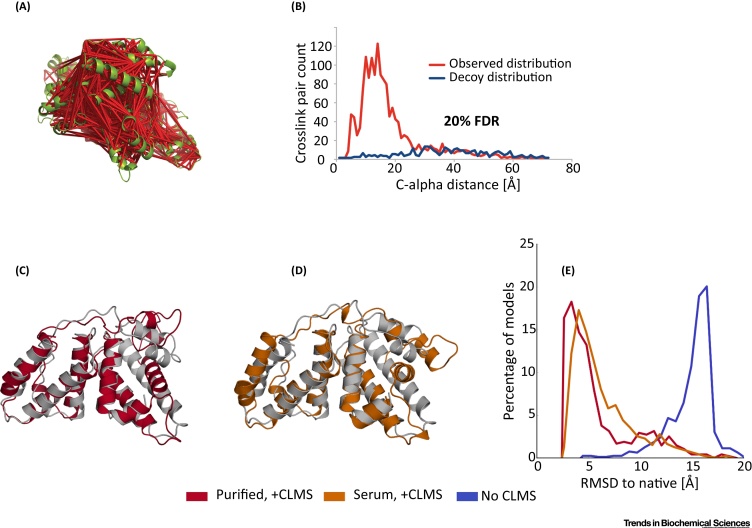
Using Photo-Crosslinking/Mass Spectrometry (CLMS) Crosslinkers for Structure Modeling. (A) Photo-crosslinking of human serum albumin (HSA) with sulfosuccinimidyl 4,4′-azipentanoate leads to 1495 links at 20% false-discovery rate. (B) The distance distribution of crosslinked residues follows a log-normal distribution. Most crosslinks are between residues with Cα distances below 20 Å. (C) The combination of high-density CLMS data with computational protein modeling is able to recapitulate the HSA domain structures. Here, we show the results for domain C of HSA. Models are shown in color, while the native structure is shown in gray. Using high density-CLMS (HD-CLMS) data from purified HSA samples leads to modeled structures with a root mean square deviation (RMSD) of 2.9 Å. (D) Using HD-CLMS data from HSA samples in blood serum leads to models with an RMSD of 3.8 Å to the native structure. (E) RMSD distribution of low-energy computed models using CLMS data from purified HSA (red), from HSA in blood serum (orange), and without CLMS data (blue). Using CLMS data shifts the RMSD distribution toward lower RMSD values. Thus, the CLMS effectively guides conformational space search and allows to sample nativelike, low-RMSD structures more frequently. Adapted from [18].

Our group recently set out to tackle the problem of crosslink sparseness by employing photoactivatable crosslinkers [18]. We demonstrated that the high density of crosslinks attainable by photo-crosslinkers surpasses a critical threshold: it enables the *de novo* reconstruction of protein structure domains, even without specialized surface distance calculation (Figure 3A–E). In our example, we were able to reconstruct the three domains of human serum albumin with an RMSD of 2.5/4.9/2.9 Å to the crystal structure. Our approach relies on three key components: (i) using the heterobifunctional crosslinker sulfosuccinimidyl 4,4-azipentanoate, (ii) an open modification-based multistep search strategy and controlled false-discovery rate estimation to identify the crosslinks, and (iii) a specialized conformational space search algorithm called contact-guided model-based search for constraint-driven *de novo* modeling 50, 51. This algorithm includes crosslink constraints in a low-resolution structural sampling phase to steer conformational space search and groups candidate structures into ‘funnels’ to build an approximate model of the energy landscape. This model is then used to allocate computational resources to promising regions in the energy landscape. The algorithm uses a specialized (flat-bottom Lorentzian) energy term to account for the case that constraints (including crosslinks) might be noisy.

Our final high-density-CLMS (HD-CLMS) data set that resulted from this study contains 1495 crosslinks (2.56 links per residue). Perhaps the most striking result of our study is that the photo-crosslinking analysis was also successful on samples in the complex, native environment of human serum albumin: human blood serum (Figure 3D). The reconstructed structure for domain C from samples in blood serum was not as accurate as from purified samples (3.8 Å RMSD from crystal structure vs. 2.9 Å). Nevertheless, the resulting structure still captured the overall correct fold.

To openly assess the generality of this approach, we participated in the 11th Community-wide Critical Assessment of techniques for protein Structure Prediction experiment (CASP11) which releases sequences of proteins with known but not publicly released structures, allowing research groups to make blind structure predictions that are independently assessed. For the first time in CASP, our group provided experimental data for CASP proteins. We recorded HD-CLMS data for four CASP proteins in a double-blind manner: the protein structures were unknown to us and unknown to the prediction groups that only had access to the protein sequences and crosslinking data for modeling. However, the data only lead to a slight improvement of the resulting models, because the four chosen proteins were very challenging to model, even for top-tier prediction groups. Nevertheless, the experiment confirmed that HD-CLMS generates distance constraints that are in good agreement with the crystal structures of the target proteins and demonstrated that it is possible to produce HD-CLMS data for proteins with unknown structure 52, 53, 54.

In CASP12, we contributed crosslink data on three target proteins, two of which form a heterodimer. For the single protein, the crosslinking data lead to a remarkable increase in modeling accuracy. The GOAL method, one of the best performing methods for *de novo* structure prediction in CASP12, improved their own blind prediction by 79% [global distance test – total score (GDT_TS) increase from 27.6 to 49.4]. The significance of this advancement is still unclear, especially as the number of test cases is still very low. Nevertheless, the CASP12 results suggest that prediction groups are increasingly able to leverage the CLMS data and we will possibly see larger and more general improvements in CLMS-assisted predictions in the future. Perhaps most importantly, the CASP experiments revealed shortcomings of the method and laid out a road map for future improvement [54].

## Current Challenges for Structural Modeling with Crosslinking Data

**Figure 4:**
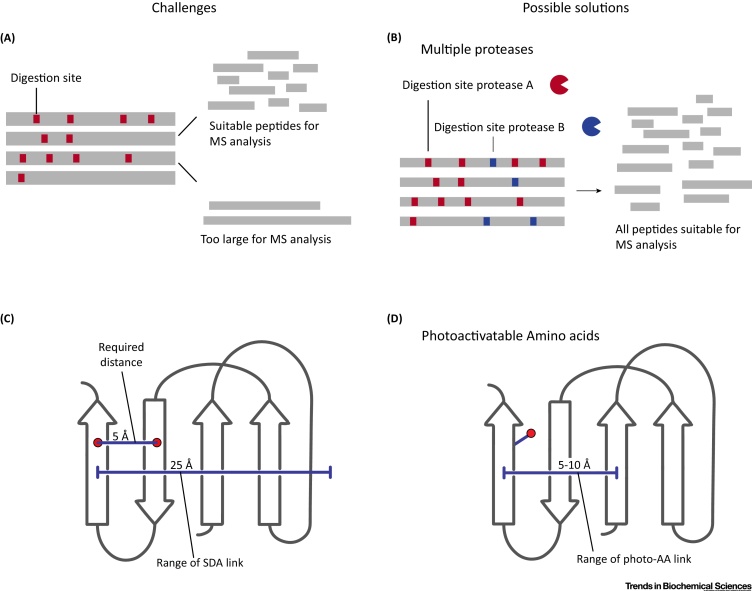
Challenges in Crosslinking/Mass Spectrometry (CLMS)-Driven Structure Determination. (A) One of the current challenges in crosslinking for structure determination is the uneven distribution of digestion sites in the protein sequence. Long-sequence stretches without trypsin digestion sites generate large peptides that are unsuitable for MS analysis. Consequently, no links can be detected in these regions. (B) Using alternative proteases or multiple enzymes for digestion could alleviate this issue by cutting these regions into smaller peptides, which can be detected in the MS. (C) Another current challenge for CLMS structure analysis is β-sheets. β-Sheets form compact structure arrangements and the distance between two β-strands is ∼5 Å. Current crosslinkers generate distance constraints of 20–35 Å [20–25 Å for sulfosuccinimidyl 4,4′-azipentanoate (sulfo-SDA)]. This is not sufficient to resolve β-sheet arrangements. (D) We speculate that using photo-amino acids could alleviate the issue, where the crosslinker formed by the side chain should lead to tighter distances constraints in the 10 Å range. Adapted from [53].

The CASP11 experiments revealed that our current experimental protocol still has open issues. In this section, we review these issues and also discuss current open questions in this field of research (see Outstanding Questions). One issue is the uneven distribution of crosslinks over the protein, which is affected by the distribution of digestion sites in the protein sequence (Figure 4A). Trypsin digestion sites are common in the proteome (trypsin cuts a protein after K and R residues; frequencies are 5.8% for K and 5.5% for R [55]) but might be unevenly distributed in the target protein. This uneven distribution results in some tryptic peptides that are either too small or too large for MS analysis, which leads to regions of the protein devoid of detectable crosslinks and therefore of structural information. A potential remedy to this issue is using alternative proteases (like Glu-C, Asp-N, and proteinase K) that target different digestion sites either alone or in combination 56, 57 (Figure 4B). Crosslinked peptides might also be missed during MS acquisition because they are of low abundance even with enrichment strategies such as size exclusion [57] or strong cation exchange chromatography 11, 58. Researchers previously improved the crosslink distribution using different and sometimes multiple crosslinker chemistries that target other residues 59, 60, 61. Using photo-crosslinkers with different chemistry could also improve the distribution of photo-crosslink data [62]. We also think that it is critical to support these experimental approaches by novel bioinformatic data analysis methods. Using multiple proteases and crosslinker chemistries will inevitably increase the complexity of the resulting MS data and careful analysis of the resulting spectra is needed to reveal more effective data analysis and acquisition methods [63].

Another issue that we often observe is the apparent lack of crosslinks in the β-sheet regions of a protein. The protein sequences did not offer an obvious explanation for this, suggesting a structural influence 52, 53. In addition, current crosslinkers might not be sufficiently informative to model β-sheet arrangements, because the crosslinkers can span over several β-strands (Figure 4C). Shorter crosslinkers that provide tighter distance constraints could be more informative and of high value in protein modeling 16, 24. A recent study suggests exactly this: Brodie *et al*. [64] combined a short-range crosslinker with discrete molecular dynamics and were able to successfully model the β-sheet-rich FK506 binding protein. Another strategy to overcome this issue is by using photoactivatable amino acids, which are incorporated into proteins during translation 65, 66 (Figure 4D). Incorporation of photo-amino acids should, in theory, not be influenced by secondary structure and therefore overcome the lack of crosslink data in β-sheets. In addition, photo-amino acids form the crosslinker themselves and therefore should result in much tighter distance constraints in the 5–10 Å range.

However, CLMS-driven hybrid structure modeling methods should be adapted to leverage crosslinking data better. To some degree, short-range crosslinkers and photo-amino acids lay on the opposite side of the spectrum than HD crosslinkers. The former set of approaches generates few, but highly informative constraints, while the latter generates many, but potentially noisy constraints. Both types of crosslinking require specialized structure modeling methods to exploit their type of crosslinking data effectively. Short-range crosslinkers might work well with methods that strictly enforce crosslinking constraints. HD crosslinking, however, might rather benefit from Bayesian techniques with fast approximations of solvent-accessible surface paths to deal with noise and to make crosslinks more informative. However, we think that combining the two approaches into a unified method would leverage all advantages that crosslinking data have to offer and might reveal minor conformational species and provide new angles to understand protein function. Another important challenge is the integration of quantitative crosslinking data to study conformational changes and dynamics with molecular dynamics or Monte Carlo simulations. Automated modeling techniques such as that presented by Ferber *et al*. [14] might play an increasingly important role in generating structural models from crosslinking studies on proteomic scale 16, 67.

## Concluding Remarks and Future Perspectives

Advances in HD crosslinking and protein modeling make this technique increasingly useful for detailed structure determination of tertiary protein structure. Further experimental method developments will aim at increasing the crosslinking yield and sequence coverage while optimizing the analysis process to reduce experimental efforts. Structural modeling needs to find ways to incorporate the increasingly complex crosslink data and model proteins larger than the current upper boundary of 100–300 amino acids. Life science researchers will need to validate these models beyond known crystal structures. Lastly, it might be a good time for the crosslinking field to consolidate and provide life scientists easy-to-use tools and best practices to establish crosslinking as an important pillar in structural biology.

Outstanding Questions Can digestion protocols using multiple proteases robustly ensure the required sequence coverage for structural studies?

Can we obtain more structural information using multiple, complementary crosslinker chemistries?

How can crosslink search software deal with the increased spectral complexity caused by multiple proteases and crosslinkers?

Can we use machine learning to improve the scoring of crosslinked peptides?

How do we increase the abundance of crosslinked peptides to enable their mass spectrometric acquisition?

How can we improve the correct site calling of photo-crosslinks?

Can the advantages of short-link crosslinks and photoactivatable amino acids (short linker length) be combined with high-density crosslinking to obtain comprehensive structural data of a protein?

How can modeling methods maximally exploit short-range crosslinking data?

How can modeling methods maximally exploit high-density crosslinking data?

Can Bayesian treatment of crosslinking constraints in structural modeling enable automated treatment of noisy and/or conflicting crosslinking constraints?

How can we integrate quantitative crosslinking data with computational protein structure modeling to model conformational changes?

How can we automate crosslink data analysis and structure modeling to enable high-throughput structure analysis studies?

How can we use crosslinking to perform *in-cell* tertiary protein structure determination?

## Footnotes

1 1997: Chemistry; structural studies on the ATP synthetase; 2003: Chemistry; structural studies on the potassium channel; 2006: Chemistry; structural studies on the eukaryotic transcription apparatus; 2009: Chemistry: structural studies on the ribosome; 2012: Chemistry: structural studies on G-protein-coupled receptors; 2017: Chemistry: development of cryo-electron microscopy for high-resolution structures.
